# Inhibition of platelet aggregation by carbon monoxide-releasing molecules (CO-RMs): comparison with NO donors

**DOI:** 10.1007/s00210-012-0732-4

**Published:** 2012-02-25

**Authors:** Stefan Chlopicki, Magdalena Lomnicka, Andrzej Fedorowicz, Elżbieta Grochal, Karol Kramkowski, Andrzej Mogielnicki, Włodzimierz Buczko, Roberto Motterlini

**Affiliations:** 1 Department of Experimental Pharmacology, Chair of Pharmacology, Jagiellonian University Medical College, Krakow, Poland; 2 Jagiellonian Centre for Experimental Therapeutics (JCET), Jagiellonian University, Krakow, Poland; 3 Pharmacodynamics, Medical University in Bialystok, Bialystok, Poland; 4 INSERM U955, Faculté de Médecine, Université Paris Est, Creteil, France

**Keywords:** CO-releasing molecule (CO-RMs), Platelet aggregation, NO donors, Human platelets

## Abstract

Carbon monoxide (CO) and CO-releasing molecules (CO-RMs) inhibit platelet aggregation in vitro. Herein, we compare the anti-platelet action of CORM-3, which releases CO rapidly (*t*_½_ 1 min), and CORM-A1, which slowly releases CO (t_½_ = 21 min). The anti-platelet effects of NO donors with various kinetics of NO release were studied for comparison. The effects of CO-RMs and NO donors were analyzed in washed human platelets (WP), platelets rich plasma (PRP), or whole blood (WB) using aggregometry technique. CORM-3 and CORM-A1 inhibited platelet aggregation in human PRP, WP, or WB, in a concentration-dependent manner. In all three preparations, CORM-A1 was more potent than CORM-3. Inhibition of platelets aggregation by CORM-A1 was not significantly affected by a guanylate cyclase inhibitor (ODQ) and a phosphodiesterase-5 inhibitor, sildenafil. In contrast, inhibition of platelet aggregation by NO donors was more potent with a fast NO releaser (DEA-NO, *t*_½_ = 2 min) than slow NO releasers such as PAPA-NO (*t*_½_ = 15 min) or other slow NO donors. Predictably, the anti-platelet effect of DEA-NO and other NO donors was reversed by ODQ while potentiated by sildenafil. In contrast to NO donors which inhibit platelets proportionally to the kinetics of NO released via activation of soluble guanylate cyclase (sGC), the slow CO-releaser CORM-A1 is a superior anti-platelet agent as compared to CORM-3 which releases CO instantly. The anti-platelet action of CO-RMs does not involve sGC activation. Importantly, CORM-A1 or its derivatives representing the class of slow CO releasers display promising pharmacological profile as anti-platelet agents.

## Introduction

Endogenous carbon monoxide (CO) formation in mammals is catalyzed by a family of heme oxygenase enzymes (HO), an inducible isoform (HO-1) and a constitutive protein (HO-2), that decompose heme into CO, ferrous iron, and biliverdin, the latter being converted to bilirubin by biliverdin reductase (Maines and Kappas 1977; Tenhunen et al. 1969). CO is recognized as a signalling molecule within the cardiovascular system being involved in the regulation of vascular tone as well as exerting anti-inflammatory, anti-apoptotic, anti-atherogenic, anti-proliferative, and cytoprotective activities (Motterlini and Otterbein 2010). The biological effects of CO, mainly manifested through vascular smooth muscle relaxation and mitigation of inflammatory processes, involve stimulation of soluble guanylate cyclase (sGC) and cyclic guanosine monophosphate (cGMP) production (Furchgott and Jothianandan 1991; Utz and Ullrich 1991), activation of calcium-dependent potassium channels (Wang and Wu 1997), stimulation of p38 mitogen-activated protein kinase (MAPK) (Otterbein et al. 2000; Otterbein et al. 2003), and direct binding to the heme moiety of structural and functional proteins such as iNOS (Foresti and Motterlini 1999; Sawle et al. 2005).

Carbon monoxide-releasing molecules (CO-RMs), of which the chemical and biochemical features have been thoroughly characterized by Motterlini and co-workers (Johnson et al. 2003; Motterlini 2007; Motterlini et al. 2003), liberate CO in biological systems providing a useful research tool for exploring the mechanism by which CO exerts its pharmacological activities (Motterlini et al. 2002). Two of these compounds, tricarbonylchloro(glycinato)ruthenium(II) (CORM-3) and sodium boranocarbonate (CORM-A1), have unique features as they are fully water-soluble and have been shown to simulate the bioactivities of gaseous CO including vessel relaxation (Clark et al. 2003; Foresti et al. 2003), protection against organ ischemia-reperfusion injury (Clark et al. 2003; Foresti et al. 2004; Guo et al. 2004), prevention of organ rejection following transplantation (Clark et al. 2003), and inhibition of the inflammatory response (Sawle et al. 2005).

Importantly, CORM-3 is a metal carbonyl complex that rapidly liberates CO in physiological buffers (half-life <1 min) (Clark et al. 2003), while CORM-A1 that does not possess a transition metal but a carboxylic moiety liberates CO at a much slower rate (half-life 21 min) under physiological conditions (Motterlini et al. 2005). Recently, we have shown that CORM-3 inhibits platelet aggregation in vitro through a mechanism that is independent of soluble guanylate cyclase activation (Chlopicki et al. 2006). The aim of the present study was to use the two mostly characterized water-soluble CO-RMs to evaluate how the different rates of CO release affect platelet aggregation in vitro and finally compare their anti-platelet profile with the one exerted by NO donors possessing various kinetics of NO release.

## Materials and methods

### Detection of CO release by assessing carbonmonoxy myoglobin formation

The release of CO from CORM-3 and CORM-A1 was assessed spectrophotometrically by measuring the kinetics of conversion of deoxymyoglobin (deoxy-Mb) to carbonmonoxy myoglobin (MbCO) as described previously (Motterlini et al. 2003). Myoglobin solutions (50 μmol/l final concentration) were prepared freshly by dissolving the protein in 0.1 mol/l phosphate buffer (pH 7.4). Sodium dithionite (0.1%) was added to convert myoglobin to deoxy-Mb prior to each experiment. CO released from CORM-3 and CORM-A1 (final concentrations: 10, 30, 40 μM) was quantified by adding aliquots of stock solutions (10 μl) of the compounds (in pure distilled water) directly to the deoxy-Mb. The kinetics of MbCO formation was quantified by measuring the change in absorbance at 541 nm (background wavelength 558 nm) for deoxy-Mb at 37°C using a spectrophotometer equipped with kinetic software package. The rates of MbCO formation were calculated and expressed as change in MbCO concentration (μM).

### Measurement of CO release from CORMs using a CO electrode

Blood specimens (20 ml) were collected on the day of experiment from healthy volunteers. Specimens were added in 5-ml volumes to TEKLAB™ blood collection tubes containing 8.75 mg of the anti-coagulant tripotassium EDTA (1.75 per ml of blood). These specimens were then gently mixed by inversion for 10 min to guarantee complete solubilizing of the tripotassium EDTA.

Each blood sample was centrifuged for 15 min at 100 × *g* at 4°C resulting in the three following layers: the inferior layer composed of red cells, the intermediate layer composed of white cells, and the superior layer made up of plasma. The plasma layer was examined for red cells. If red cells were present, the sample was re-centrifuged for an additional 5 min. The plasma layer was removed and retained as platelet rich plasma (PRP). The remaining blood specimen was then centrifuged at 1,500 × *g* for 20 min to collect the platelet poor plasma layer.

The release of CO from CORM-A1 and CORM-3 in buffer or PRP was detected using a prototype electrode purchased from World Precision Instrument (WPI; Stevenage, Herts, UK) and used as previously described (Motterlini et al. 2005). This CO electrode is a membrane-covered amperometric sensor which has been designed on a basic operating principle similar to the nitric oxide (NO) sensor. The CO sensor can be connected to the WPI ISO-NO Mark II meter for detection of the current signals providing that the poise potential is set to a different value (900 mV for CO as opposed to 860 mV for NO). The electrode was immersed into either 1 ml of buffer solution or 1 ml of PRP and was equilibrated at 37°C for 30 min prior to addition of CO-RMs. Once equilibrated, the electrode was zeroed, and CO-RM (0.3 or 3 mM) was added. Readings were taken until the chart reached the maximal CO release, and data are expressed as an average of three independent experiments.

### Measurement of carbonmonoxy hemoglobin in the whole blood upon exposure to CO-RMs

CORM-3 or CORM-A1 was added to the whole blood, and after 2 or 10 min of incubation, carbonmonoxy hemoglobin (COHb) content in the blood was analyzed using automatic blood gases analyzer (NovoMedica). After 2 and 10 min of incubation at 36°C, blood samples (100 μl) were placed in a CO-oximeter (Ciba-Corning 270, Siemens), where the blood was hemolyzed with UV and COHb level was determined. Before first and after last run of blood with CO-RM, three runs with control blood were performed. The zero point was set before first run and after automatically, every 30 min with Wash/Zero solution (Bayer). The calibration was performed once in a month with CO-OX Slope solution (Bayer), according to producer instruction.

### Platelet aggregation assay 

#### Isolation of human platelets 

Venous blood was obtained from human volunteers at the University Hospital Blood Bank Centre. Volunteer donors had not taken any medicines for the preceding 2 weeks. Blood was collected into vials containing sodium citrate (3.2%, 9:1 *v*/*v* or 3.8%, 10:1 *v*/*v*) as an anti-coagulant agent.

For the platelet aggregation in full blood, venous blood samples were diluted with 0.9% NaCl (1:1 *v*/*v*). To obtain PRP, blood was centrifuged at 250 × *g* for 20 min. Washed platelets (WP) were obtained from PRP which were washed twice in PGI_2_-containing phosphate buffered saline (PBS) according to the method of Radomski et al. (1988) and finally suspended (2 × 10^8^ platelets/ml) in Ca^2+^-free PBS containing 0.1% albumin and 0.1% glucose. Contamination of neutrophils in WP was less than 1/10^8^ (Chlopicki et al. 2004).

#### Platelet aggregation assay in humans

Aggregation of blood platelets in the whole blood was assessed using Chronolog aggregometer (Chrono-log Corp., USA) by measurements of electrical impedance according to the method described by Cardinal and Flower (1980), while in PRP and WP, it was assessed by measurements of optic transmittance according to the method described by Born (1967).

Whole blood was equilibrated for 13 min, and then, CORM-A1, CORM-3, or VEH (0.9% NaCl) were added. Collagen was added after further 2 min of incubation. The dose of collagen was chosen to cause 50% of full aggregation (EC_50_). The stirrer speed was set at 800 rpm. Changes in impedance were registered 6 min after stimulation with collagen. The maximal extension of the aggregation curve at sixth minute was expressed as a percentage of control value.

PRP (500 μl) was equilibrated for 2 min at 37°C with continuous stirring at 1,100 rev/min and then stimulated with collagen to cause aggregation. At the beginning of each experiment, concentrations of collagen that induced sub-maximum aggregation response were determined. These were in the range of 0.5–1.5 μg/ml. CORM-3, CORM-A1, RuCl_3_, inactive form of CORM-A1, DEA-NO, PAPA-NO, DETA-NO, DPTA-NO, and SNAP were added usually 2 min before stimulation of platelets with collagen unless longer incubation was indicated. In some experiments, ODQ (10 μM) or sildenafil (100 nM) was added 1 min prior to the addition of CORMs or NO donors.

WP (500 μl) was equilibrated for 2 min at 37°C with continuous stirring at 1,100 rev/min in PBS containing CaCl_2_ and MgCl_2_ both at a concentration of 1 mM and then stimulated with thrombin to cause aggregation. At the beginning of each experiment, concentrations of thrombin that induced sub-maximum aggregation response were determined. These were in the range 15–20 mU/ml. CORM-3, CORM-A1, DEA-NO, PAPA-NO, DETA-NO, and DPTA-NO were added 2 min before stimulation of platelets unless longer incubation time was indicated. In some experiments, ODQ or sildenafil was added 1 min prior to the addition of CORMs or NO donors.

### Reagents and drugs

Collagen was obtained from Chrono-Par (USA), thrombin from Biomed (Poland), DEA-NO (half-life ~2 min at 37°C and pH = 7.4), PAPA-NO (half-life ~15 min), DPTA-NO (half-life ~3 h), DETA-NO (half-life ~20 h), and ODQ were purchased from Cayman (USA). Ru(CO)_3_Cl(glycinate) (CORM-3) and sodium boranocarbonate (Na[H3BCO2H] or CORM-A1) were synthesized as previously described (Clark et al. 2003; Foresti et al. 2003) (Motterlini et al. 2005). The inactive form of CORM-A1 which does not release CO (iCORM-A1) was prepared as described previously (Motterlini et al. 2005). Ruthenium chloride (RuCl_3_) was used as a negative control for CORM-3.

### Statistical analysis

All calculations were performed with GraphPad Prism software. Values of (lg) IC_50_ were calculated by sigmoidal dose–response curve fitting (variable slope, and fixed bottom and top values to 0 and 100, respectively). Least squares method was used unless the residuals did not follow normal distribution; in this case, robust fit was used. However, this last method did not report confidence intervals for estimated parameters. Comparison of active form of CORMs with inactive counterparts and influence of ODQ or sildenafil on action of CORMs or NO donors were assessed by Mann–Whitney test or with Kruskal–Wallis test followed by Dunn’s multiple comparisons. Results of whole blood aggregation were compared by unpaired *t* test.

## Results

### Characteristics of CO release from CORM-3 and CORM-A1

**Fig. 1:**
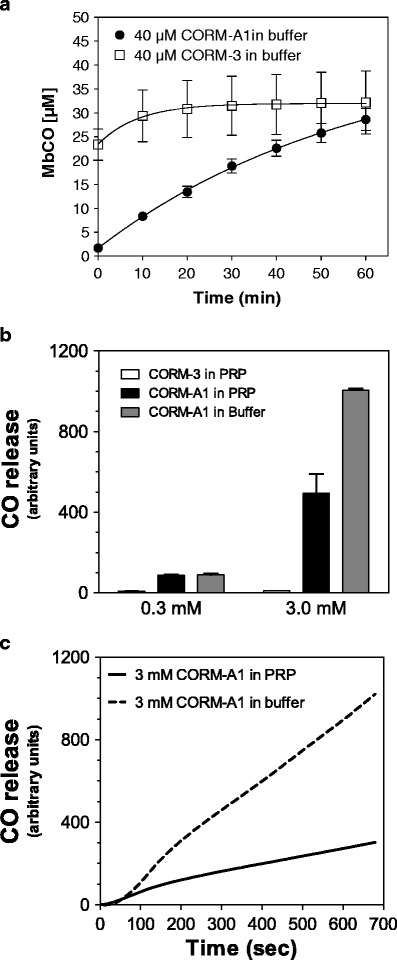
Time course of MbCO formation from CORM-3 and CORM-A1 in buffer (**a**) and comparison of CO-releasing capacity from CORM-3 in PRP and CORM-A1 in PRP and Krebs buffer (**b**). The time course of releasing CO by CORM-A1 in buffer and PRP (**c**)

As detected by the myoglobin assay (Fig. 1a), CORM-3 released CO almost instantly in buffer (half-life <1 min at 37°C, pH 7.4), while CORM-A1 released CO at much slower rate (half-life ≈ 21 min at 37°C, pH 7.4). Although the CO electrode detected the release of CO from CORM-A1 in PRP, which occurred in a slow fashion as previously described in buffer, this device was not sensitive enough to detect measurable amounts of CO from CORM-3 (Fig. 1b). Scavenging of CO by plasma constituents interacting with CORM-3 could represent one possible reason for the failure of the CO electrode to detect any CO in PRP. Alternatively, CORM-3 releases CO effectively only in the presence of an avid acceptor, as in the case of reduced myoglobin. In fact, as exemplified in Fig. 1c, there was a striking difference in CO detection also from CORM-A1 between PRP and buffer solutions showing much less CO being measured in PRP (Fig. 1c). When CO-RMs were incubated in the whole blood for few minutes, we found that COHb increased from 1.5 ± 0.17% (control) to 2.6 ± 0.15% with 1 mM CORM-3, while 300 μM CORM-A1 did not significantly changed this parameter (1.76 ± 0.17%).

### Effects of CORM-3 and CORM-A1 on human platelet aggregation in PRP, WP**,** and whole blood

**Fig. 2:**
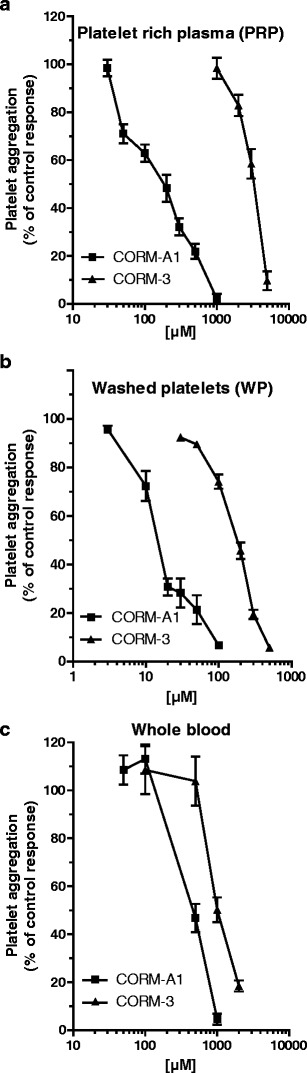
Concentration-dependent anti-aggregatory effects of CORM-3 and CORM-A1 in human PRP (**a**) (*n* = 4–52), human WP (**b**) (*n* = 4–39), and human whole blood in vitro (**c**) (*n* = 3–9)

As shown in Fig. 2a, when added to human PRP, CORM-A1 or CORM-3 inhibited platelet aggregation induced by collagen in a concentration-dependent manner. However, CORM-A1 was effective in the micromolar concentrations range, while CORM-3 was effective only at millimolar concentrations (IC_50_ = 160.3 μM and 3170 μM, 95% CI from 2900 to 3464 μM, for CORM-A1 and CORM-3, respectively). Similarly, the inhibitory effect of CORM-A1 on thrombin-induced platelet aggregation in WP exceeded that of CORM-3 (Fig. 2b). Even though approximately ten times lower concentrations of both CORM-3 and CORM-A1 were needed to inhibit washed human platelets as compared to PRP, CORM-3 was more than a tenfold weaker anti-platelet agent than CORM-A1 in WP preparation (IC_50_ = 13.82 μM and 171.9 μM for CORM-A1 and CORM-3, respectively, Table 1). Also, the inhibitory effect of CORM-A1 on collagen-induced platelet aggregation in vitro human whole blood exceeded that of CORM-3 (Fig. 2c), though the difference in potency was approximately by twofold (IC_50_ = 467.45 μM and 1004.16 μM for CORM-A1 and CORM-3, respectively; Table 1). Thus, even though the potency of CORM-A1 and CORM-3 differed in PRP, WP, and the whole blood, CORM-A1 was a more potent anti-platelet agent than CORM-3 in human PRP, WP, as well as in the whole human blood.

**Table 1:** IC_50_ values for inhibition of platelets aggregation by CO-RMs and NO donors in whole blood, platelet rich plasma, and washed platelets. All values are interpolated from concentration-dependent curves with the exception of IC_50_ for DETA-NO in PRP which was extrapolated from the concentration-dependent curve since even at the concentration of 30 mM DETA-NO inhibited platelet aggregation by approximately 40%

		Inhibition of platelet aggregation (IC_50_)		
CO-RMs	*t*_½_	Whole blood (μM)	Platelet rich plasma (μM)	Washed platelets (μM)
CORM-3	<1 min	1004.2	3170.0	171.9
CORM-A1	21 min	467.4	160.3	13.8
NO donors				
DEA-NO	2 min	N.D.	0.24	0.022
PAPA-NO	15 min	N.D.	0.48	0.051
DPTA-NO	3 h	N.D.	8.33	0.37
DETA-NO	20 h	N.D.	38.9	6.5

*N.D.* not determined

**Fig. 3:**
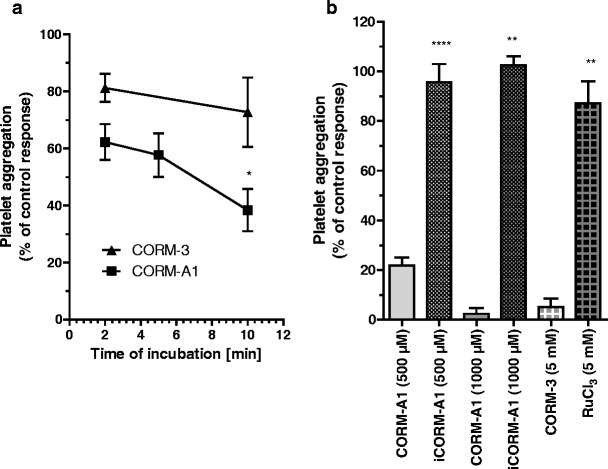
**a** Influence of incubation time on the anti-aggregatory effect of CORM-3 and CORM-A1 in PRP (**a**) (*n* = 7–14), 2 mM CORM-3 and 100 μM CORM-A1 were used, respectively). **b** Lack of the anti-aggregatory effect of inactive forms of CO-RMs in PRP (*n* = 5–46). One asterisk and three asterisks denote statistical significance vs. standard incubation of 2 min

For the comparison of the anti-platelet activities of CORM-3 and CORM-A1 in PRP and WP, both compounds were incubated for 2 min prior to the addition of collagen (PRP) or thrombin (WP) (Fig. 2a, b). If the incubation time for CORM-3 was prolonged to 5 or 10 min prior to the stimulation of platelet aggregation either in PRP (Fig. 3a) or WP (data not shown), the anti-platelet activity of CORM-3 was not at all potentiated. In contrast, if CORM-A1 was pre-incubated for 5 or 10 min prior to the stimulation of platelet aggregation in PRP (Fig. 3a), the anti-platelet effect of CORM-A1 was more accentuated. Similarly, prolongation of CORM-A1 incubation in WP accentuated its anti-aggregatory effects (8.167 ± 1.01% and 25.17 ± 1.96% after 2 and 5 min of incubation with 30 μM of CORM-A1, respectively; *p* < 0.001, *n* = 5–6). The difference in activity of CORM-A1 but not CORM-3 with the prolonged pre-incubation is fully compatible with the instant release of CO from CORM-3 (half-life <1 min at 37°C, pH 7.4) and the slower rate of CO release from CORM-A1 (half-life ≈ 21 min at 37°C, pH 7.4). The respective negative controls used for CORM-3 (RuCl_3_) and CORM-A1 (iCORM-A1) that do not possess CO-dependent activity were inactive as anti-platelet agents (Fig. 3b).

### Effects of various NO donors on human platelet aggregation in PRP and WP 

**Fig. 4:**
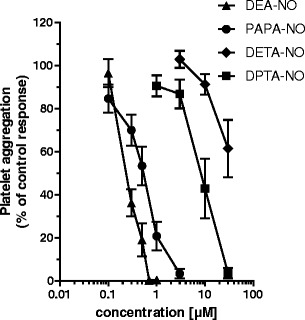
Concentration-dependent anti-aggregatory effect of NO donors in PRP

The comparison of anti-platelet activity of various NO donors (DEA-NO, PAPA-NO, DETA-NO, and DPTA-NO) in PRP is shown in Fig. 4. Both in PRP and WP, the NO donor with the shortest half-life was a more potent inhibitor of platelet aggregation than NO donors possessing a slower rate of NO release. In PRP, DEA-NO, (*t*_½_ = 2 min at 37°C and pH = 7.4), PAPA-NO (*t*_½_ = 15 min), DPTA-NO (*t*_½_ = 3 h), and DETA-NO (*t*_½_ = 20 h) inhibited platelets with an IC_50_ of 0.24 μM, IC_50_ = 0.48 μM, IC_50_ 8.33 μM, and IC_50_ = 38.9 μM, respectively. A similar order of potency for these four NO donors was observed in WP (Table 1).

### Differential involvement of soluble guanylate cyclase in the anti-platelet action of CO-RMs and NO donors

**Fig. 5:**
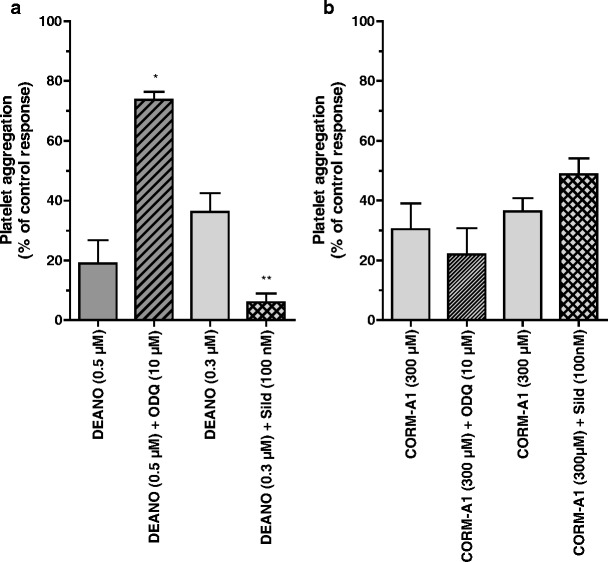
Effect of ODQ (10 μM) (*n* = 11–14) and sildenafil (100 nM) (*n* = 7–12) on the anti-aggregatory action of DEA-NO (**a**) (*n* = 3–16) and CORM-A1 (**b**) (*n* = 7–14) in PRP

As shown in Fig. 5a, an inhibitor of soluble guanylate cyclase activity (ODQ) reversed the inhibition of platelet aggregation induced by DEA-NO, while sildenafil profoundly enhanced the inhibition of platelet aggregation induced by DEA-NO, both in PRP (Fig. 5a) and in WP (data not shown). ODQ and sildenafil displayed similar effects on platelet response to other NO donors (e.g., SNAP; data not shown).

In contrast, in the presence of an inhibitor of soluble guanylate cyclase activity (ODQ), the effect of CORM-A1 was fully preserved either in collagen-stimulated platelets aggregation in PRP (Fig. 5b) or in thrombin-induced platelet aggregation in WP (data not shown). In the presence of a selective inhibitor of phosphodiesterase-5 (sildenafil), the anti-platelet effect of CORM-A1 in PRP (Fig. 5b) and in WP (data not shown) was not potentiated.

## Discu**s**sion

In the present work, we evaluated the time- and concentration-dependent anti-platelet effects of two water-soluble CO-releasing agents (Motterlini et al. 2002; Motterlini et al. 2003; Motterlini et al 2005). These two compounds are (1) a metal carbonyl complex that rapidly liberates CO (CORM-3) and (2) a novel releaser of CO that does not contain a transition metal (sodium boranocarbonate, CORM-A1) and generates CO at a much slower rate under physiological conditions. Using three different in vitro systems for platelet aggregation (PRP, WP, and whole blood), we demonstrated a superior anti-platelet profile of activity for CORM-A1 compared to CORM-3. Indeed, the anti-aggregatory effects of CORM-A1 in PRP and whole blood were achieved in a concentration range that does not significantly affect the formation of COHb. Furthermore, prolongation of pre-incubation of platelets with CORM-A1 but not CORM-3 potentiated the observed effect suggesting that effective anti-aggregatory action by micromolar concentration of CORM-A1 may be achieved through a gradual liberation of CO over time. Taking into account that CORM-3 elicited prompt and rapid vasodilatory effects in vitro and in vivo whereas CORM-A1 promoted prolonged vasodilation and milder hypotensive effect in vivo (Motterlini et al. 2005), one can speculate that CORM-A1 could afford an anti-aggregatory effect in vivo without a hypotensive effect and possibly without a toxic effect related to increased COHb formation which might compromise oxygen delivery to tissues. Predictably, exogenous NO affords simultaneously hypotensive and anti-aggregatory effects on platelets, which are both mediated by activation of sGC. In the present work, the pharmacological effects of both CO-RMs on platelets do not appear to involve activation of soluble sGC supporting our previous work on CORM-3 and pointing out that the identification of a specific target for CO in platelets remains a challenge (Chlopicki et al. 2006).

The biological activities of CO and NO are frequently compared. Here, we underscore clear differences on the anti-aggregatory properties of CO-RMs and NO donors that could reflect the biological activity of endogenous NO and CO on platelet aggregation. Endogenous NO instantly produced by the vascular endothelium can be stimulated by increased blood flow, shear stress, or an agonist, and the rapid kinetics of endogenous NO release has been detected in biological systems (Kalinowski et al. 2003). On the other hand, our body is continuously exposed to small quantities of CO produced endogenously during the degradation of heme by heme oxygenase enzymes (HO-1 and HO-2), while a greater quantity of CO is produced during stress conditions upon the induction of inducible HO-1. In view of the data presented here, we can speculate that to mimic an effective anti-aggregatory action of CO on platelets, slow but sustained kinetics of CO release is preferable as indicated by the significantly more potent anti-aggregatory effect obtained with CORM-A1.

It has been consistently shown that sGC is strongly activated by NO and to a lesser extent by CO (Friebe et al. 1998; Hoenicka et al. 1999; Stone and Marletta 1998). Here, we found that in contrast to NO donors, the anti-aggregatory activity of CORM-A1 was not mediated by sGC. Indeed, in our experiments, ODQ, a potent and selective inhibitor of sGC (Garthwaite et al. 1995), completely reversed the anti-platelet effect of DEA-NO, while it was ineffective against CORM-A1. Furthermore, sildenafil, a phosphodiesterase (PDE)-5 inhibitor, failed to potentiate the effect of CO liberated by CORM-A1 in human platelets. At the same time, sildenafil amplified the anti-aggregatory effect of NO released by DEA-NO. Previously, we demonstrated that CORM-3 inhibited platelets by a sGC-independent mechanism, while others had shown that high concentrations of gaseous CO (100%) inhibited aggregation via activation of sGC (Brune et al. 1990). Here, using ODQ, a selective inhibitor of sGC, and using sildenafil, a PDE-5 inhibitor that inhibits degradation of cGMP, we provide evidence that inhibition of platelet aggregation by physiologically relevant concentrations of CO liberated by CORM-A1 was mediated by a sGC-independent mechanism. Apparently, in platelets as well as other tissues, sGC does not appear to be a primary target for the bioactivity of micromolar concentration of CO. Apart from sGC (Christodoulides et al. 1995; Furchgott and Jothianandan 1991; Hussain et al. 1997), there are a number of other possible targets for CO such as calcium-activated potassium channels (Wang and Wu 1997), cytochrome P450 (Coceani et al. 1997), mitochondrial respiratory chain (Lo Iacono et al. 2011), or p38 MAPK (Amersi et al. 2002; Zhang et al. 2003). The role of each of these potential targets responsive to CO in mediating the anti-aggregatory effects of CO-RMs remains to be fully investigated.

Despite the fact that the specific target(s) for the anti-aggregatory action of low concentrations of CO in platelets remain(s) unknown, our data point out that the use of CO-RM may represent a novel approach for selective and effective inhibition of platelets in vivo. We previously demonstrated that in contrast to NO and PGI_2_, CO effectively inhibited platelet aggregation even when platelets were excessively activated (Chlopicki et al. 2006). These results suggest that CO may play a role of a retaliatory mediator that comes into play when NO and PGI_2_ are insufficient to overcome excessive platelet activation, and this can be mimicked by CO-releasing agents.

The comparison between the effect of CO-RMs and NO donors on inhibition of platelet aggregation offers some additional points of discussion that should be taken into consideration. First of all, it is clear that all NO donors tested are more potent inhibitors of platelet aggregation than either CORM-3 or CORM-A1. Secondly, the slow CO releaser (CORM-A1, *t*_½_ = 21 min) is in average 15 times more potent than the fast CO carrier (CORM-3, *t*_½_ < 1 min) in inhibiting aggregation, and this is true both in PRP and WP. In the whole blood, CORM-A1 is still more potent than CORM-3 by approximately twofold. Interestingly, an opposite profile is observed for the NO donors since DETA-NO, which has a half-life of approximately 25 min, is 150 times less potent than the fast NO donor DEA-NO (*t*_½_ = 1 min). Notably, the intriguing observation of our experiments is that the anti-aggregation effect of both NO donors and CO-RMs is ten times more pronounced in washed platelets than in PRP. Although the reduced effect on platelet aggregation in plasma may be predicted in the case of NO donors, this is not so intuitive and is rather unexpected for CO-releasing compounds. In fact, it is well established that NO, apart from interacting strongly with ferrous (Fe^2+^) and ferric (Fe^3+^) metal centers, has a preferential reactivity with thiols and cysteine moieties present in proteins (Stamler et al. 2001; Stamler et al. 1992). Although this post-translational modification is illustrated in human red blood cells by S-nitrosylation of hemoglobin which has profound physiological implications on oxygen transport and delivery (Jia et al. 1996), the interaction of NO with cysteines in plasma is best exemplified by the rapid formation of S-nitrosoalbumin and possibly other stable circulating S-nitrosoproteins (Stamler et al. 2001; Stamler et al. 1992). The fact that in order to have the same degree of inhibition of aggregation, a higher concentration of NO donors is required in platelets present in plasma (PRP) compared to washed platelets which suggests that NO liberated from these agents is partially scavenged by the plasma components, possibly proteins rich in cysteines.

In the case of CO, it is more difficult to explain why CO-RMs elicit a reduced inhibition of platelet aggregation in plasma compared to washed platelets since only the presence of proteins containing ferrous iron (Fe^2+^) or other transition metals with a specific redox state could act as effective scavengers of CO. From the analysis of our results on the differential effects of CORM-3 and CORM-A1 in PRP, washed platelets, and in whole blood, it appears that the kinetics of CO release is an important factor in determining the potency of CO-RMs as anti-aggregatory agents. Our data using a sensitive CO electrode also reveal that indeed the total amount of CO liberated from CORM-A1 in PRP is much higher than the amount released from CORM-3; however, both the amount and the rate of CO release from CORM-A1 are markedly reduced in plasma compared to PBS especially when very high concentrations of CORM-A1 are used (3 mM). These data do not exclude the possibility that transition metal-containing proteins or enzymes present in plasma could act as potential scavengers of CO thus diminishing the potency of CORM-A1 in PRP. The general perception is that once present in the blood stream, CO would bind irreversibly to hemoglobin and eliminated through the lung by respiration, but a revision of this concept may be required as plasma components (i.e,. metal-containing proteins) may act either as potential targets of CO or carriers of CO to be transported either in circulating cells or tissues (Boczkowski et al. 2006; Foresti and Motterlini 2010). On the other hand, in the case of CORM-3, the increase in COHb is not substantial if one considers that heme concentration in human blood hemoglobin is in the order of 10 mM, and thus, 1 mM CORM-3 should potentially generate 10% COHb. The fact that much less is detected with 1 mM CORM-3 indicates that the total transfer of CO from the metal carbonyl to the heme in hemoglobin is not occurring or is somehow prevented by other factors. In the case of CORM-A1, the lower formation of COHb suggests that micromolar concentrations of CORM-A1 may achieve effective anti-platelet effect without a significant COHb formation.

In conclusion, CORM-A1 that releases CO at a slower rate is a superior inhibitor of platelet aggregation than the fast CO carriers such as CORM-3. Furthermore, our results underscore the importance of both the chemical features of compounds releasing gaseous molecules and the kinetics of the gaseous mediator release that determine their anti-platelet efficacy. There is a clear difference between NO donors and CO-releasing molecules as regard to their mechanisms and pharmacodynamic characteristics of anti-platelet action that might help to determine an intracellular target for anti-platelet activity of low micromolar concentration of CO that is not shared by NO. Finally, our work points out that CORM-A1 displays promising anti-aggregatory activities, and thus, this compound or similar slow-releasers of CO should be exploited therapeutically further as an anti-thrombotic drug in vivo.

## Abbreviations

CORM-3: Tricarbonylchloro(glycinato)ruthenium(II)
CORM-A1: Sodium boranocarbonate
DEA-NO: Diethylammonium (*Z*)-1-(*N*,*N*-diethylamino)diazen-1ium-1,2-diolate
DETA-NO: (*Z*)-1-[*N*-(2-aminoethyl)-*N*-(2-ammonioethyl)amino]diazen-1-ium-1,2-diolate
DPTA-NO: (*Z*)-1-[*N*-(3-aminopropyl)-*N*-(3-ammoniopropyl)amino]diazen-1-ium-1,2-diolate
iCORM-A1: CORM-A1 deactivated by nitrogen bubbling in acidified solution
L-NAME: *N*-nitro-l-arginine methyl ester
ODQ: 1H-[1,2,4]Oxadiazolo[4,3-a]quinoxalin-1-one
PAPA-NO: (*Z*)-1-[*N*-(3-aminopropyl)-*N*-(*n*-propyl)amino]diazen-1-ium-1,2-diolate
SNAP: *S*-nitroso-*N*-acetyl penicillamine
